# In‐Foam Bioprinting: An Embedded Bioprinting Technique with Self‐Removable Support Bath

**DOI:** 10.1002/smsc.202300280

**Published:** 2024-01-31

**Authors:** Elias Madadian, Hossein Ravanbakhsh, Francesco Touani Kameni, Maedeh Rahimnejad, Sophie Lerouge, Ali Ahmadi

**Affiliations:** ^1^ Department of Mechanical Engineering École de technologie supérieure Montreal H3C 1K3 Canada; ^2^ Biomaterials and Biofabrication Laboratory Centre de recherche du Centre hospitalier de l’Université de Montréal (CRCHUM) Montreal H2X 0A9 Canada; ^3^ Department of Biomedical Engineering The University of Akron Akron OH 44325 USA; ^4^ Department of Pharmacology and Physiology Université de Montreal Montreal H3T 1J4 Canada

**Keywords:** albumin foams, biofabrication, chitosan hydrogel, embedded bioprinting, tissue engineering

## Abstract

The emergence of embedded three‐dimensional (3D) bioprinting has revolutionized the biofabrication of free‐form constructs out of low‐viscosity and slow‐crosslinking hydrogels. Using gel‐based support baths has limitations including lack of proper oxygenation and nutrition and complications with bath removal. Herein, a novel‐embedded 3D bioprinting technique is developed with an albumin foam support bath as a promising substitute. The proposed technique, in‐foam bioprinting, offers excellent printability and convenience in bath removal while providing cells with easy access to oxygen and nutrients. The foam‐based support bath is characterized through foam stability and rheological tests. The bubble size in the foam is measured to study the change in the structure of the bath due to the coalescence of the bubbles over time. Free‐form structures are successfully 3D printed with thermoresponsive chitosan‐based bioinks to demonstrate the capability of the in‐foam bioprinting technique. The viability of bioprinted fibroblast L929 cells is studied over a seven‐day period, showing high cell viability of over 97%, which is attributed to the abundance of oxygen and nutrition in the foam support bath. Importantly, in‐foam bioprinting is beneficial for biofabricating large samples with a long printing time without jeopardizing cell viability.

## Graphical abstract

A novel embedded bioprinting modality is introduced by employing a self‐removable and nonliquid support bath: albumin foam. This support material offers a biocompatible environment that is enriched with nutrients and well‐oxygenated. These unique features lead to more versatility in the bioprinting of large organs with prolonged biofabrication time without compromising the viability of embedded cells.

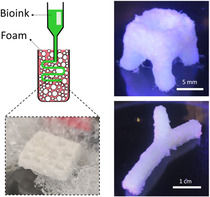

## 1 Introduction

Three‐dimensional (3D) bioprinting is the method of fabricating cell‐laden biological constructs in a layer‐wise manner.^[^ ^1^ ^]^ Owing to its simplicity, extrusion bioprinting, which works based on the programmed deposition of cell‐laden bioinks on a build plate, is the most prevalent and inexpensive technique among various modalities of bioprinting.^[^ ^2^, ^3^ ^]^ One of the critical bottlenecks for the development of extrusion bioprinting is the paucity of biocompatible, yet printable bioinks.^[^ ^4^ ^]^ Specifically, many biocompatible hydrogels do not possess sufficient mechanical and rheological properties to be 3D bioprinted.^[^ ^5^ ^]^ A strategy to address the abovementioned limitation of extrusion bioprinting is using a support bath for bioprinting low‐viscosity materials to maintain high shape fidelity.^[^ ^6^ ^]^ The method, generally known as embedded 3D bioprinting, was introduced in 2011^[^ ^7^ ^]^ and later expanded through different terminologies: self‐healing hydrogels,^[^ ^8^ ^]^ granular hydrogels,^[^ ^9^ ^]^ and freeform reversible embedding of suspended hydrogels.^[^ ^10^ ^]^

Embedded 3D bioprinting has outperformed conventional extrusion methods in terms of printing resolution, free‐form printing, and the versatility of compatible bioinks.^[^ ^11^ ^]^ An important aspect of this technique is the choice of material for the support bath. Various materials, such as Pluronic F127,^[^ ^12^ ^]^ gelatin,^[^ ^13^ ^]^ Carbopol,^[^ ^14^, ^15^ ^]^ hyaluronic acid,^[^ ^16^ ^]^ agarose,^[^ ^17^ ^]^ gellan gum,^[^ ^18^ ^]^ xanthan gum,^[^ ^19^ ^]^ Laponite nanoclays,^[^ ^20^ ^]^ and poly(ethylene oxide)^[^ ^21^ ^]^ have been utilized as support baths so far. Although embedded 3D bioprinting in its current format is conducive to biofabricating superlative constructs out of low‐viscosity bioinks, there are several considerations and limitations when gel‐based support baths are utilized. Primarily, the limited availability of dissolved oxygen and nutrient diffusion in the bioprinted cell‐laden constructs before the bath removal may cause necrosis or hypoxia‐induced apoptosis, which is a major obstacle to keeping the cells viable during long gelation processes, e.g., thermal gelation.^[^ ^22^, ^23^ ^]^ Furthermore, some bath removal mechanisms can be detrimental to cell viability and structural fidelity, especially when lowering the temperature or mechanical agitation is the only way to remove the bath.^[^ ^24^ ^]^ Moreover, removing gel support from cavities and confined parts of the printed structure can be cumbersome. Scant attention has been devoted to rectifying such deficiencies associated with embedded 3D bioprinting. Therefore, there is a need for developing nutrient‐enriched support baths that provide abundant oxygen to the cells and can be conveniently removed to overcome the abovementioned issues.

Albumin, a well‐known foaming agent,^[^ ^25^, ^26^, ^27^ ^]^ has been previously used as a biocompatible and biodegradable material.^[^ ^28^ ^]^ It is a natural water‐soluble protein present in human blood, bovine serum, and chicken egg white.^[^ ^29^ ^]^ Mechanical mixing of albumin solution denatures the protein structure and creates long protein chains exposed to the surrounding environment. This phenomenon allows hydrophilic and hydrophobic groups to trap the adjacent gas phase (i.e., surrounding air) within the solution and subsequently, create foam when mechanically mixed.^[^ ^30^, ^31^ ^]^ After foaming, the liquid phase of the foam flows downward due to its higher density.^[^ ^30^ ^]^ We hypothesize that by employing amphipathic molecules, such as egg white albumin as the support bath in bioprinting, the gradual coalescence of bubbles would be advantageous to further simplify the removal of the support bath thus extracting the printed structures after the bioprinting process. Other possible advantages of albumin foam as the support bath over conventional support baths, such as gelatin slurry, can include the ease of access to the surrounding oxygen in the gas phase rather than the limited dissolved oxygen in conventional baths^[^ ^32^ ^]^ and the possibility of providing cells with nutrients during bioprinting via enriching the foam solution with cell culture media.

**Figure 1:**
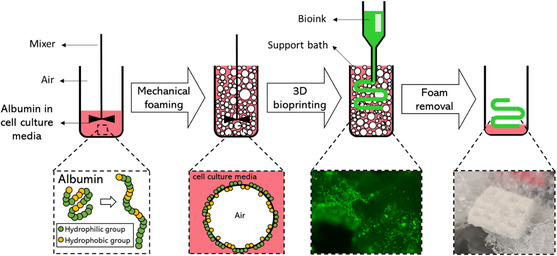
Schematic illustration of the in‐foam bioprinting process. The chicken egg white albumin foam was prepared by mechanical mixing of albumin powder in cell culture media. The produced foam was then employed as the support bath for bioprinting cell‐laden constructs.

In this work, we introduced in‐foam bioprinting (**Figure** **1**), a method of biofabrication based on using albumin foam as the support bath for bioprinting free‐form structures out of low‐viscosity slow‐crosslinking hydrogels in a biocompatible setting. We tested different concentrations of albumin with various foaming times to determine the optimum experimental conditions for creating the foam. Rheological and physical characterization of the foam revealed that the bubbles in the foam play an important role in making a self‐removable substrate for bioprinting. Compared to conventional support baths, we have shown that a unique advantage of using foam‐based support baths in embedded bioprinting is the capability of bubbles to merge after bioprinting, resulting in a sacrificial support bath. As proof of concept, the in‐foam bioprinting technique was used for bioprinting various structures with chitosan‐based thermosensitive hydrogels, that are not printable using conventional bioprinting modalities due to their low viscosity and long gelation time. The cell compatibility of the process was demonstrated using L929 fibroblasts as a model cell. The results support the fact that in‐foam bioprinting can be readily employed for fabricating complex cell‐laden structures with applications in tissue engineering and therapeutic technologies.

## 2 Results and Discussion

### 2.1 Rheology

**Figure 2:**
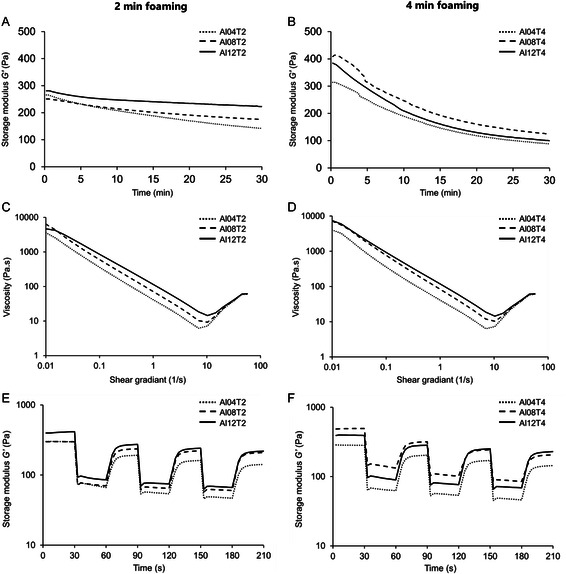
Rheological properties of albumin support baths (AlαTβ: α% w v^−1^ albumin foamed for β minutes): A,B) evolution of the storage modulus (*G*′) as a function of time at 37 °C; C,D) shear‐thinning behavior: viscosity as a function of shear rate; E,F) recovery test: storage modulus of albumin support baths during various cycles of strain at 37 °C (30 s rest at 1% strain, 30 s under shear at 100% strain) (mean values of triplicates are plotted).

An ideal support bath must be stable over the period of bioprinting. It must be able to recover its original state and rheological properties, such as storage modulus, after the bioprinter nozzle moves inside it. Albumin support bath groups (i.e., foams with different concentrations of albumin) were first characterized to assess their stability at the bioprinting temperature, i.e., 37 °C, via time sweep tests. Their shear thinning and recovery behaviors at 37 °C were also studied. The main results of rheological tests are presented in **Figure** **2**, for a foaming time of 2 min (left) or 4 min (right), and described in detail in the following sections.

#### 2.1.1 Time Sweep

Figure 2A shows the storage modulus over time for various baths mechanically foamed for 2 min. The storage modulus slowly decreased over time for all the foam compositions. However, it was more stable for concentrations of 12% and 8% w v^−1^ albumin compared to 4% w v^−1^ albumin. Within 30 min, *G*′ decreased from ≈280 Pa to 220 Pa, i.e., 21% decrease for albumin 12%, from ≈250 Pa to 180 Pa, i.e., 28% decrease for albumin 8%, and from ≈270 Pa to 140 Pa, i.e., 48% decrease for albumin 4%. Such decrease in storage moduli can be explained by bubbles merging and coalescence over time. When the foaming time increased to 4 min, higher *G*′ was observed for all formulations, as shown in Figure 2B. Longer foaming time resulted in unfolding more albumin proteins in the liquid phase of the foam. This phenomenon resulted in stiffer bubbles and increased *G*′ for samples foamed for 4 min. The collapse of stiffer foam over time resulted in a sharper decrease of storage modulus in the 4‐min foamed albumin. A decrease of ≈65% in the storage modulus for these samples indicates that although increasing the foaming time boosts the storage modulus, the stability of the storage modulus is negatively affected, which is not preferable in embedded bioprinting. It was concluded that 2 min of mechanical foaming is sufficient for making stable foam.

#### 2.1.2 Shear‐Thinning Viscosity

The support bath must maintain a shear‐thinning behavior in order not to hinder the movement of the bioprinting needle.^[^ ^22^ ^]^ The viscosity of the support bath was studied as a function of applied shear rate. All albumin foam groups showed a general shear‐thinning behavior, as demonstrated in Figure 2C,D. Such shear‐thinning trend is similar to that of the previously developed gel‐based support baths.^[^ ^33^, ^34^ ^]^ An increase in viscosity (i.e., shear‐thickening) was observed when the applied shear stress was larger than 10 s^−1^, which is attributed to the instability of the rheometer in high frequencies. Changing the foaming time did not have any noticeable effect on the general shear thinning behavior of the foam, while a lower concentration of albumin resulted in a lower viscosity. Such observation is in accordance with the fact that low concentrations of albumin yield more dispersed molecules and thus less intermolecular interaction between solute, i.e., albumin, and solvent, i.e., cell culture media, molecules, which led to lower viscosity.^[^ ^35^ ^]^ However, the foaming time, which does not change the content of the albumin solution, has minimal effect on the viscosity.

#### 2.1.3 Recovery

An important property of support bath materials is their capability to recover the initial state when the needle passes through and deposits the filaments. Figure 2E,F shows the ability of the albumin support baths mechanically foamed for 2 and 4 min, to recover throughout cyclic deformation. Among the foam groups, albumin 8% mechanically foamed for 2 min showed the best cyclic recovery with only 31.0% ± 5.5% drop of storage modulus after several cycles, while other formulations showed more than 40% decrease in the storage modulus (Figure 2E). This observation is justified by the fact that using lower concentrations of albumin, results in higher liquid drainage of the foam.^[^ ^36^ ^]^ The liquid drainage results in a drier foam which exhibits poor recovery. In contrast, the abundance of entangled albumin chains in 12% albumin concentration resulted in a stiffer foam (as discussed in Section 3.1.1), which is not capable of recovering its shape. As a result, there is an optimum albumin concentration that yields a support bath with acceptable recovery. Such rapid recovery to an initial state ensures that the extruded bioink is soundly embedded.^[^ ^13^ ^]^ Similar to other support baths, a recovery of ≈80% of the initial moduli is considered acceptable for embedded bioprinting.^[^ ^37^ ^]^

### 2.2 Bubbles Size

**Figure 3:**
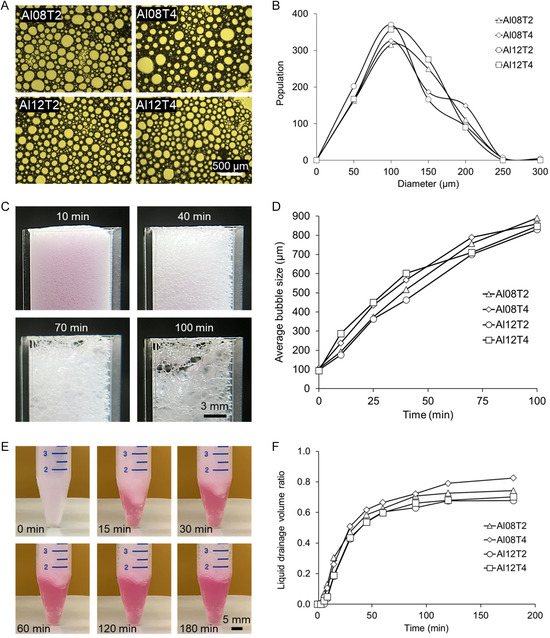
Physical characterization of albumin foam in cell culture media. A) Optical microscope images of bubbles immediately after preparing the foams. B) Size distribution of the bubbles. C) Images of bubbles merging over time in 8% albumin foam. D) Average bubble size for various albumin foams over time. E) Images of 8% albumin foam in a 15 mL conical centrifuge tube while the phase separation between liquid and gas develops. F) Quantification of foam stability based on the liquid drainage volume ratio over time.

As shown in Video S1 (Supporting Information), the bubbles in the foam merge over time and gradually disappear as the phase separation between liquid and gas occurs. **Figure** **3**A shows microscopic images of bubbles captured from various foams, and Figure 3B shows a histogram of the diameter of bubbles. The size distribution is similar for all foam compositions and foaming times where the majority of bubbles have a diameter of 50 to 150 μm. As shown in Figure 3C,D, the average bubble size increases over time until the large bubbles start bursting. Such behavior of the albumin foam addresses a persistent issue with bath removal in conventional embedded bioprinting methods. This feature gives the user the capability to bioprint delicate structures without jeopardizing the fidelity of the structure during crosslinking and bath removal.

### 2.3 Foam Stability

The coalescence of bubbles after mechanical foaming results in the gradual phase separation within the foam. Albumin concentration and foaming time are the two main factors investigated in this study as they can influence the rate of phase separation. As shown in Figure 3E,F and Video S2 (Supporting Information), the liquid phase of all the study groups gradually flowed downward over time. Although the groups with a lower concentration of albumin tend to be less stable, as mentioned in Section 3.1.3, the difference is not statistically significant. It has been shown that the stability of the albumin foam is significantly related to the pH of the solution and additives such as polysaccharides can be used as stabilizers to enhance the albumin foam stability.^[^ ^38^, ^39^ ^]^ In the case of bioprinting large structures with several hours of printing time, stabilizers might be recommended to be added to the albumin solution to ensure the stability of the bath during the whole bioprinting procedure. To confirm the effectuality of the polysaccharides on the stability of the foam and the size of the bubbles over time, 2% sodium alginate and 8% albumin solution were foamed and observed over 2 h (Supporting Information Video S1‐B with blue food coloring). At 100 min, the average size of the bubbles was measured at 323 ± 9 μm, which is 2.75 times smaller than that of the 8% albumin foam without the stabilizer (Video S1‐A, Supporting Information). However, since self‐removability feature of this method depends on the coalescence of bubbles, in the ideal scenario, the stability of the foam should be adjusted based on the printing time. This adjustment will prevent any possible negative effect that gravity might cause.

### 2.4 Printability

**Figure 4:**
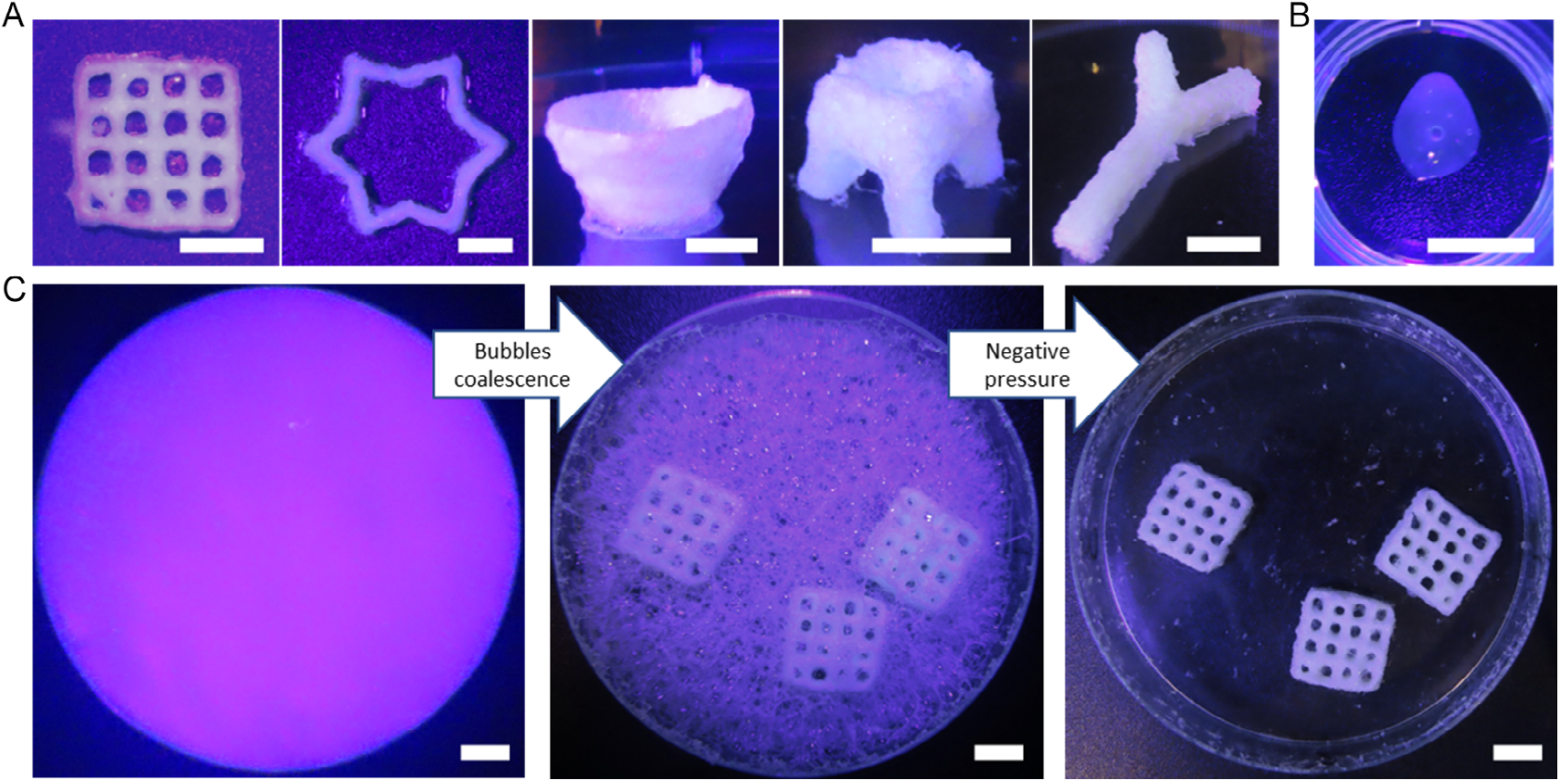
In‐foam printability: A) various chitosan structures fabricated in 8% albumin foam. B) A chitosan grid structure printed without foam. The print has failed due to poor shape fidelity. C) Pictures of samples in the foam right after printing, phase change of foam over time, and after removing the foam with the use of negative pressure. Scale bars are 1 cm.

The feasibility of the in‐foam embedded printing process was examined by 3D printing several constructs made of slow‐crosslinking chitosan or chitosan–collagen hydrogels. As shown in **Figure** **4**A, the in‐foam printing method was successfully used to fabricate various constructs from grid patterns to free‐form conical structures (Supporting Information Videos S3: 3D printing with RegenHU bioprinter and S4: 3D printing using BioX bioprinter). Figure 4A also demonstrates an intrinsic disadvantage of albumin foam, which is the invisibility of the bioprinting process due to light diffraction. To highlight the importance of using foam as the support bath, we tried to print a grid structure without the foam (Figure 4B). This failed printing job indicated the essential role of support baths in printing chitosan and chitosan–collagen.

After bioprinting, the two phases of the foam separated over time, while the samples slowly gelled at 37 °C (Figure 4C). The coalescence of bubbles in the foam led to the gradual disappearance of the foam (Supporting Information Video S5) without jeopardizing the viability of embedded cells. Since the foam was intrinsically made of air bubbles, most of the remaining foam (if any) was readily removed using negative pressure (Supporting Information Video S6). This convenient support bath removal is a paramount advantage of the in‐foam printing method. Due to its negative electrical charge, traces of albumin could however be observed on the printed structures, making a rough surface on the positively charged chitosan samples. Such a rough surface could be beneficial to cell adhesion and growth on the samples.^[^ ^40^ ^]^ However, albumin protein residues could also influence the biocompatibility of the printed structures, and will require, in the future, the use of human albumin instead of chicken egg white albumin. The printability (Pr) number^[^ ^26^ ^]^ of the printed grid structures is calculated as 0.87 ± 0.03. It is noteworthy that the remaining albumin residues on the samples adversely impact the characterization calculations for in‐foam bioprinted structures using methods such as the Pr number (Pr = *L* ^2^/16*A*, *L*: perimeter, and *A*: area of squares). However, the overall resolution of the printed samples, either with chitosan bioink or chitosan–collagen bioink, was visually acceptable and comparable to the other embedded bioprinting methods.^[^ ^22^ ^]^

### 2.5 Cell Viability

**Figure 5:**
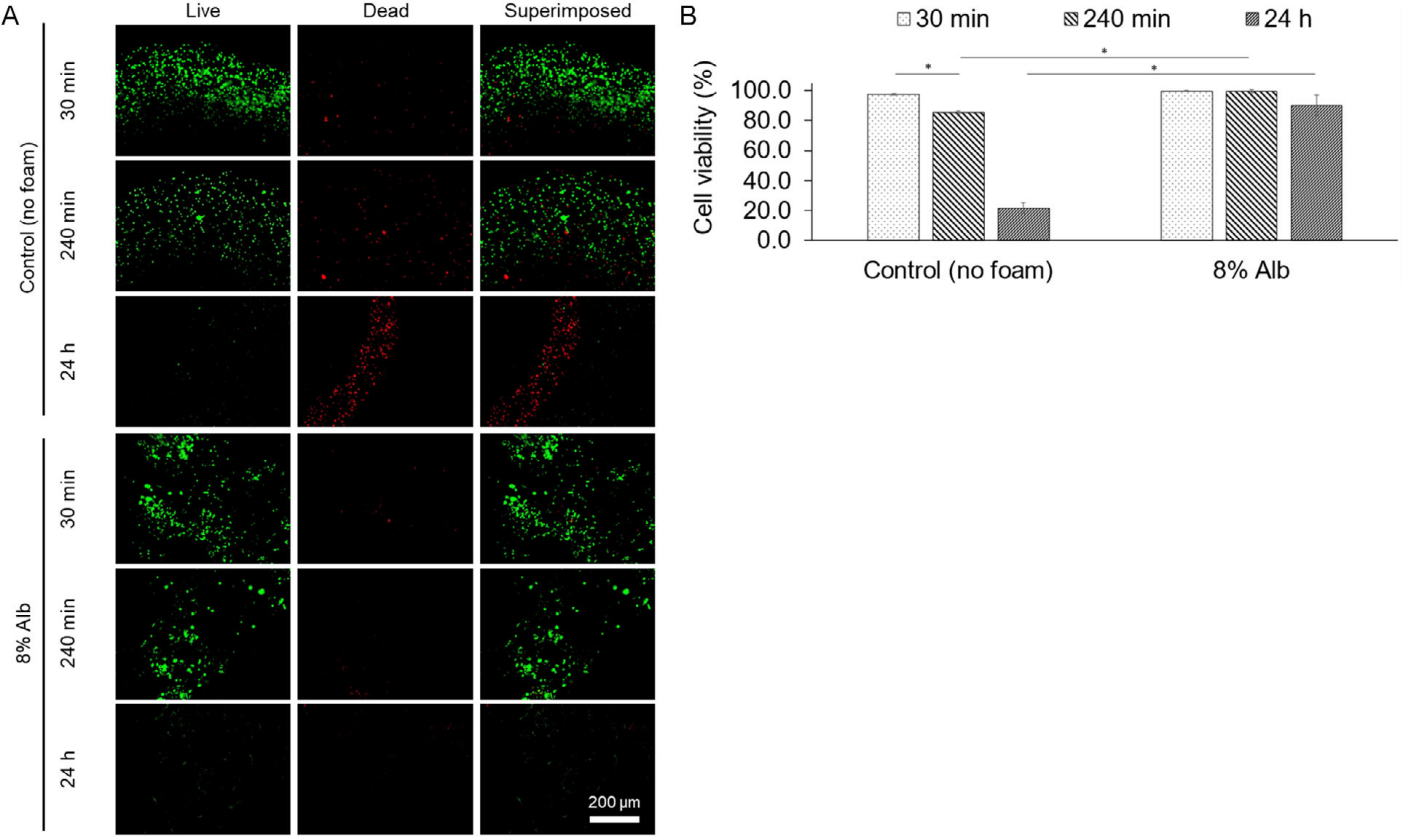
Effect of time before adding cell culture media (and removing the support foam) on the cell viability in bioprinted chitosan–collagen grid constructs, compared to control (without support bath). A) Live/dead fluorescence images; B) quantified cell viability values. (Alb: albumin, mean ± SD, *n* = 3, *p < 0.05).

Cell viability studies were first performed to understand how long the cells can stay viable in chitosan–collagen constructs when bioprinted in the nutrient‐enriched foam bath. A delay between bioprinting and foam removal is needed to ensure the samples are well crosslinked before adding cell culture media, otherwise, the bioprinted constructs would collapse. Cell‐laden grid constructs were bioprinted inside and outside of the 8% albumin foam, and the cell culture media were added at 30 min, 240 min, or 24 h, after removing the excessive bath. For control groups that solely investigate the effect of the foam presence on the cell viability, bioprinted out of the foam, phosphate‐buffered saline (PBS) 1× was immediately added before adding media to avoid drying off. **Figure** **5**A shows the live/dead fluorescent images of samples on day 1. As shown in Figure 5B, cell viability was significantly decreased for control samples when incubated for 240 min without media. As expected, the majority of cells died after 24 h of starvation. It was observed that the cells in the control samples were more concentrated in the peripheral area of the hydrogel. The percentage of viable cells in the in‐foam bioprinted samples was not significantly influenced by this delay, even when incubated for 24 h in the foam without adding additional cell culture media. However, at the 24 h timepoint, a significant percentage of the cells have left the structure and the size of the live cells significantly decreased, which can be a sign of initiation of apoptosis.^[^ ^41^ ^]^ The observations of this study support our hypothesis on the advantages of a nutrient‐enriched foam bath in keeping the cells viable during and after bioprinting for 24 h. Also, when a foam support bath is used, the bioprinted cells have access to proper oxygenation throughout the structure, preventing hypoxia. While no standard gel support bath exists for benchmarking our cell viability results, more targeted investigations into cellular oxygenation can elucidate the potential advantages offered by the presence of gas‐phase oxygen compared to the limited dissolved oxygen levels in traditional gel‐based support baths. The capability of keeping the cells viable for an extended time alludes to another advantage of the in‐foam method when the bioprinting procedure takes several hours, especially when slow‐crosslinking bioinks or large constructs such as full organs are bioprinted.^[^ ^42^ ^]^

**Figure 6:**
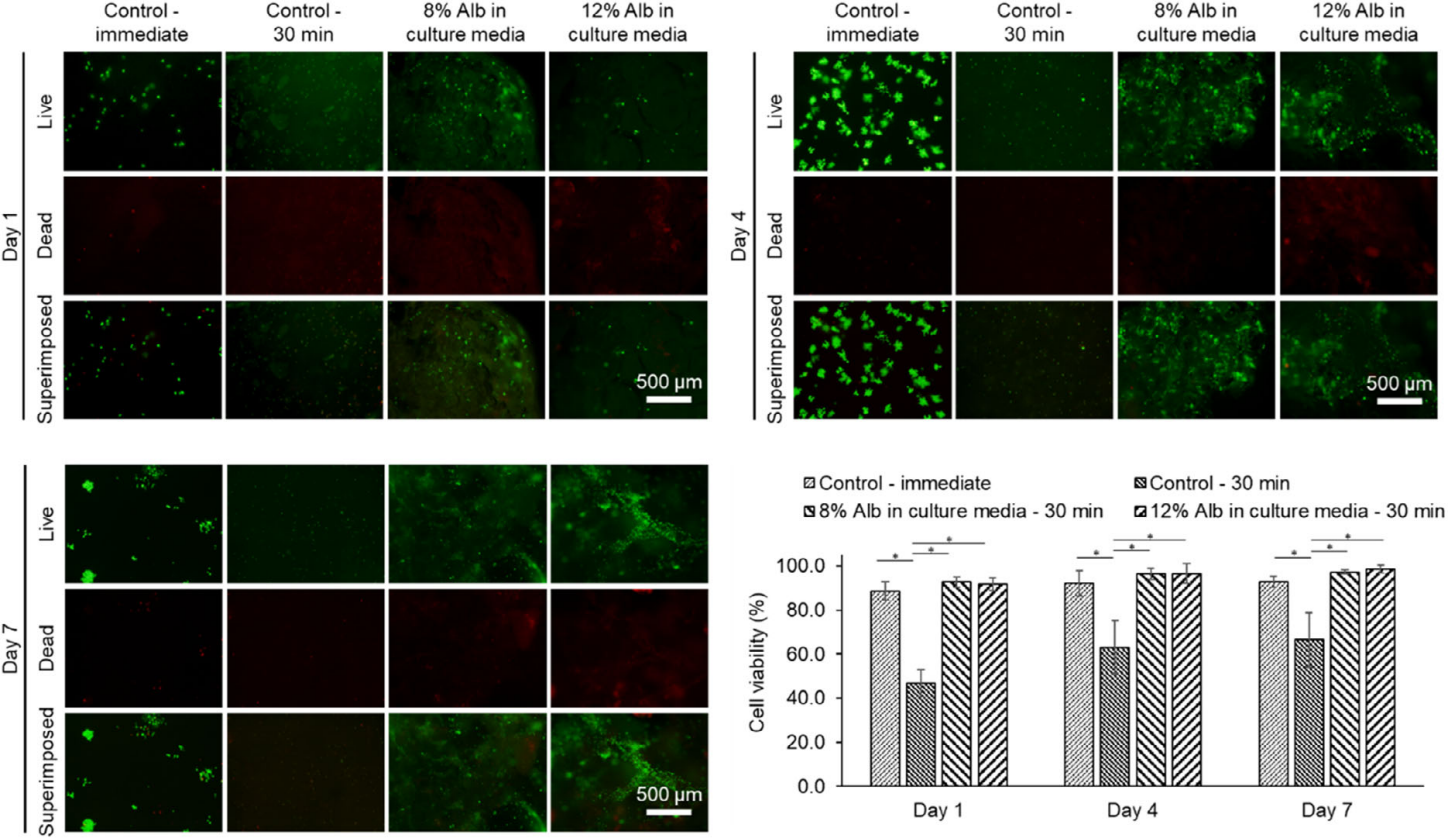
Live/dead fluorescence microscopy images and cell viability results for control and in‐foam bioprinted samples for a 7‐day cell culture. Using albumin foam as the support bath in bioprinting has significantly enhanced cell viability compared to the control ‐ 30 min group. (Alb: albumin, mean ± SD, *n* = 3, *p < 0.05).

In the second step, 7‐day period cell viability study was performed to evaluate the effect of albumin concentration (8% and 12% in completed Dulbecco's Modified Eagle Medium (DMEM)) on the viability of cells embedded in chitosan–collagen structures. The foam was removed, and cell culture media were added 30 min after bioprinting. Two control groups bioprinted without foam were tested, where the cell culture media were added either immediately (positive control) or 30 min after bioprinting (negative control). Analyzing the live/dead fluorescent microscopy images on days 1, 4, and 7 (**Figure** **6**) revealed that the in‐foam bioprinted samples have significantly higher cell viability compared to the negative control group. We observed the concentration of albumin had minimal effect on cell viability. The cell viability rate for the positive control is not significantly different from that of the in‐foam bioprinted group. However, adding media immediately after bioprinting, which was done for the positive control, is not always feasible for two reasons: 1) many bioinks do not crosslink immediately, thus a wait time is needed before adding media and 2) bioprinting large constructs can be time‐consuming and adding media needs to be delayed until the whole construct is biofabricated. Moreover, a support bath is needed in many cases to get acceptable printing resolution, as exemplified here by the poor aspect of the control grid structure printed without foam (see Figure 4B). This observation exclusively echoes the biocompatibility of our in‐foam bioprinting method and its potential in adding more flexibility and versatility to conventional bioprinting methods. The cell viability range for the in‐foam bioprinting method is comparable to other embedded bioprinting methods.^[^ ^10^, ^11^, ^43^ ^]^

## 3 Conclusion

In this study, in‐foam bioprinting, a novel embedded bioprinting modality was propounded based on utilizing a nonliquid support bath, albumin foam. Our support bath offers a biocompatible environment with a number of distinct characteristics. Since the foam is intrinsically made of bubbles, the bioprinted cells are not prone to face low oxygen levels during biofabrication and gelation time. The abundance of cell culture media and other nutrients in the foam holds promise for high cell viability. In addition, the foam bath can be gradually removed after bioprinting without the intervention of any external mechanism that might negatively affect the shape fidelity of printed structures. The in‐foam bioprinting method is reliable and effective for bioprinting low‐viscosity hydrogels with either a short or long gelation time without compromising cell viability. Additionally, using this method, large structures with a prolonged printing time can be biofabricated. While our results demonstrate the feasibility and potential of the in‐foam bioprinting approach, further studies are required to investigate the use of other cell types to study the functionality of the cells, as well as other foaming agents as the support bath or the addition of foam stabilizers to enhance the stability of the foam for embedded bioprinting. Moreover, the use of the foam to investigate the applicability of this method for bioprinting hydrogels with other crosslinking methods, such as ionically crosslinking hydrogels, needs further attention. This bioprinting technique is suitable for the biofabrication of free‐form constructs with applications in tissue engineering and precision medicine.

## 4 Experimental Section

4.1

4.1.1

##### Bioink Preparation

Two bioinks were tested in this study, namely chitosan and chitosan–collagen physical hydrogels, the composition of which is presented in **Table** **1**. Both bioinks form low‐viscosity solutions at room temperature and crosslink when the temperature is raised to 37 °C. Chitosan bioink was prepared by mixing chitosan solution and a gelling agent following the previously‐published protocol.^[^ ^44^ ^]^ Briefly, shrimp shell chitosan powder (ChitoClear, HQG110, Primex, Siglufjordur, Iceland) with a molecular weight of 155 kDa and a degree of deacetylation of 83% was dissolved in hydrochloric acid (0.1 m, HCL‐Fisher Scientific) using a mechanical mixer for 4 h to achieve a homogenous chitosan solution. The solution was then autoclaved to ensure aseptic conditions for cell studies. The gelling agent used in this study was a mixture of β‐glycerol phosphate (Sigma‐Aldrich, Oakville, ON, Canada) and sodium hydrogen carbonate (MP Biomedicals, Solon, OH, USA) dissolved in Milli‐Q water and sterilized by filtration. Since the chitosan gelation mechanism is affected by the basicity of the gelling agent, pH measurements were conducted immediately before mixing the components using a pH meter (LAQUAtwin, Horiba Advanced Techno, Kyoto, Japan). The pH of the gelling agent was adjusted to 8.0 via dropwise addition of HCl (0.1 m). Chitosan solution and the gelling agent were mixed using two syringes connected by a luer lock immediately before bioprinting. For preparing chitosan–collagen bioink, the gelling agent was first added to a collagen type I solution (Corning, Glendale, AZ), which was previously dissolved in acetic acid, prior to its mixing with the chitosan solution. The final bioink was composed of 2% w v^−1^ chitosan and 1% w v^−1^ collagen as briefed in Table 1.

**Table 1:** Composition of chitosan and chitosan–collagen bioinks

	Chitosan [% w v^−1^]	β‐glycerol phosphate [m]	Sodium hydrogen carbonate [m]	Collagen type I [% w v^−1^]
Chitosan	2.0	0.1	0.075	–
Chitosan–collagen	2.0	0.1	0.075	1.0

##### Foam‐Based Support Bath Preparation

Albumin solution was made by adding albumin powder from chicken egg white (A5253, Sigma‐Aldrich, USA) in concentrations of 4%, 8%, and 12% w v^−1^ to either deionized (DI) water or DMEM with fetal bovine serum (FBS, 10%) and then stirred at 600 rpm for 4 h. The solution was then mechanically foamed using a hand‐held mixer (TM‐300HMCN, Toastmaster Hand Mixer) for 2 or 4 min to obtain a homogenous foam.

##### Rheology

Albumin solutions (4%, 8%, and 12% w v^−1^) in DI water were mechanically foamed for 2 and 4 min. Rheological characterization of the albumin foams was carried out using an Anton Paar rheometer (Physica MCR 301, Germany) with concentric cylinder geometry (CC10/T200) and a 1 mm gap. Various tests, explained in the following subsections, were conducted to characterize the physical and rheological properties of the foams.

Time sweep: The storage modulus (*G*′) and loss modulus (*G*″) of the foams with different concentrations (4%, 8%, and 12% w v^−1^) and foaming time (2 and 4 min) were measured using time sweep tests for 30 min at 37 °C, using the oscillatory mode in the linear viscoelastic region, at a constant shear strain of 1% and constant frequency of 1 Hz to assess mechanical stability of the foam during the bioprinting process.

Recovery: Cyclic recovery tests at 37 °C were performed to verify the self‐recovery properties of the foams when the nozzle moves in the foam bath. The storage modulus of the foams was measured during various cycles mimicking the bioprinting process: 1) pre‐printing (30 s at 1% strain), 2) printing (sudden increase to 100% strain for 30 s), and 3) post‐printing (back to 1% strain).

Viscosity: The viscosity of the support bath at 37 °C was assessed using rotational rheometry tests by altering the applied shear rate from 0.01 to 100 s^−1^ to verify the shear thinning behavior of the support foam. The graph was plotted on a logarithmic scale.

##### Bubble Size

Albumin foams with the same concentrations and mechanically foaming times tested in the rheology section were prepared to characterize the air bubbles within the foams. However, due to the observed poor recovery of 4% albumin foam, this study group was not further characterized. A thin layer of foam samples was collected on a petri dish and observed with an optical microscope (AmScope, United Scope LLC, Irvine CA). Three images from random locations of the samples were taken using a high‐resolution microscope camera (MU1803‐HS, AmScope, United Scope LLC, Irvine CA). The diameter of 900 bubbles from each sample was measured using the AmScope image processing software (United Scope LLC, Irvine CA) to investigate the effect of albumin concentration and the mechanical foaming time on the initial bubble size.

The thin layer of the albumin foam used for measuring the initial bubble size could not be observed to study the coalescence of the bubbles over time since the samples dried off after some time. Therefore, bulks of foam samples were transferred to ultraviolet (UV) quartz cuvettes (Sigma‐Aldrich, Oakville, ON, Canada) using a syringe and imaged from the transparent side using the macro mode of a camera (SX740 HS, Canon, Tokyo, Japan). The samples were imaged every 10 s for a total time of 2 h. Between 200 and 500 randomly selected bubbles were measured at certain time points using the AmScope software to investigate the trend of bubbles diameter change over time.

##### Foam Stability

The stability of albumin foam groups (i.e., foams with different concentrations of albumin) was determined based on the rate of phase separation in the foam. For this purpose, the same study groups used in bubble size measurements were prepared and transferred to tubes(15 mL). The samples were placed on a hot plate at 37 °C and videoed for 3 h. The level of precipitated liquid was measured at certain time points using the AmScope software to quantify the foam stability over time.

##### Cell Culture

Mouse fibroblast L929 cells were cultured in a humidified incubator (37 °C and 5% CO_2_) in DMEM supplemented with FBS (10% v v^−1^) and penicillin/streptomycin (1% v v^−1^). The cells were trypsinized (Trypsin 0.05%/ EDTA) and passed to a new flask at 80% confluency. The cell culture media were changed every 3 days to ensure sufficient and consistent nutrients were provided to the cells. For bioprinting, cell solution (100 μL) was prepared so that a final concentration of 5 million cells mL^−1^ was obtained in the bioink. The cell solution was added to the gelling agent syringe and encapsulated in the hydrogel during the two‐syringe mixing procedure detailed previously.^[^ ^22^ ^]^ The cell‐laden bioink was then transferred to the bioprinter cartridge for biofabricating in‐foam constructs.

##### Bioprinting Setup

Bioprinting in albumin foam was performed using two different bioprinters: an extrusion‐based 3Ddiscovery bioprinter (RegenHU, Villaz‐St‐Pierre, Switzerland) with a plunger printhead and a BioX bioprinter (Cellink, Gothenburg, Sweden) with a pneumatic printhead. Cell viability studies were conducted using the 3Ddiscovery bioprinter as it provides aseptic conditions, while printability experiments were performed using both 3Ddiscovery and BioX bioprinters to demonstrate the compatibility of the in‐foam bioprinting method with different settings. For the plunger‐based bioprinter, the feed rate and filament thickness were adjusted on 7 mm s^−1^ and 0.4 mm, respectively, to ensure decent printing resolution can be achieved.^[^ ^22^ ^]^ For the pneumatic bioprinter, the printing speed and the pressure were set at 12 mm s^−1^ and 8 kPa, respectively. The bioprinting process was conducted using a stainless steel 1‐inch long 21G blunt needle (McMaster‐Carr, Elmhurst, IL, USA). While the bioink cartridge was at room temperature during bioprinting, the foam was kept at 37 °C to accelerate chitosan gelation.^[^ ^45^ ^]^ A sterilization process with ethanol and overnight UV exposure was followed to ensure the bioprinter and the experiment environment were in aseptic condition for cell studies. Immediately after printing, the in‐foam bioprinted samples were transferred to a humidified incubator with 5% CO_2_. After 24 h, the samples were washed with PBS 1×, and cell culture media were added. The media were changed every other day to ensure sufficient nutrient were accessible to the cells.

##### Cell Viability

Live/dead assays were performed to determine the effect of two parameters on cell viability: 1) incubation time before adding media and 2) albumin concentration in the foam support bath. First, the bioprinted samples (control and in‐foam) were incubated for 30 min, 240 min, or 24 h, then the cell culture media were added. It should be noted that the control samples were kept in PBS 1× before adding media to avoid drying off. On day 1 after bioprinting, the samples were stained and imaged using a fluorescent microscope to assess the effect of delay in adding media on the cell viability. For studying the effect of albumin concentration, 1, 3, and 7 days after bioprinting, the cell culture media were washed off from the bioprinted samples using PBS 1×. Calcein, AM (Invitrogen, Life Technologies, Carlsbad, CA, USA) at a concentration of 2 μM and Ethidium Homodimer‐1 (EthD‐1, Invitrogen, Life Technologies, Carlsbad, CA, USA) at a concentration of 5.5 μM in DEMEM serum free were used as per the manufacturer's protocol to stain live and dead cells, respectively for 45 min. After staining, the samples were washed with PBS 1×, placed between two microscope cover slips, and imaged using an inverted fluorescent contrast microscope (Leica DMIRB, Microscope Central, Feasterville, PA, USA). Three samples were prepared for each study group, and three images were captured at random locations of each sample. Subsequently, by normalizing green (live cells) and red (dead cells) channels using a grayscale filter, and proper thresholding of contrast and brightness levels the cell viability rate was calculated using ImageJ through measuring the projected area of red and green signals.^[^ ^46^ ^]^ The cell viability percentage was calculated as:

(1) $$\text{Cell viability \%}=\left(\frac{\text{live cells}}{\text{live cells}+\text{dead cells}}\right)\times 100$$

##### Statistical Analysis

Analysis of variance was used to implement the statistical analysis of data. In all tests, p‐values lower than 0.05 were considered statistically significant. To compare every two groups of data, Tukey post hoc analysis was implemented. All the results were reported as mean values ± standard deviations, and all the tests were done in triplicate.

## Conflict of Interest

The authors declare no conflict of interest.

## Supporting information

Supplementary Material

## Data Availability

The data that support the findings of this study are available from the corresponding author upon reasonable request.
